# Unveiling the potential of *A. fabrum* and γ-aminobutyric acid for mitigation of nickel toxicity in fenugreek

**DOI:** 10.1038/s41598-024-61894-7

**Published:** 2024-05-14

**Authors:** Subhan Danish, Ghulam Sabir Hussain, Muhammad Baqir Hussain, Abdallah M. Elgorban, Rahul Datta

**Affiliations:** 1 Department of Soil Science, Faculty of Agricultural Sciences and Technology, Bahauddin Zakariya University, Multan, Punjab Pakistan; 2 Department of Agronomy, Faculty of Agricultural Sciences and Technology, Bahauddin Zakariya University, Multan, 66000 Pakistan; 3 Department of Soil and Environmental Sciences, Muhammad Nawaz Sharif University of Agriculture Multan, Multan, Punjab Pakistan; 4 Department of Botany and Microbiology, College of Science, King Saud University, P.O. 2455, 11451 Riyadh, Saudi Arabia; 5 Department of Geology and Pedology, Faculty of Forestry and Wood Technology, Mendel University in Brno, Zemedelska 1, 61300 Brno, Czech Republic

**Keywords:** Chlorophyll contents, Fenugreek, Gas exchange attributes, Nickel, Rhizobacteria, γ-Aminobutyric acid, Plant sciences, Plant stress responses, Abiotic

## Abstract

Nickel (Ni) is a heavy metal that adversely affects the growth of different crops by inducing oxidative stress and nutrient imbalance. The role of rhizobacteria (RB) is vital to resolve this issue. They can promote root growth and facilitate the uptake of water and nutrients, resulting in better crop growth. On the other hand, γ-aminobutyric acid (GABA) can maintain the osmotic balance and scavenge the reactive oxygen species under stress conditions. However, the combined effect of GABA and RB has not been thoroughly explored to alleviate Ni toxicity, especially in fenugreek plants. Therefore, in the current pot study, four treatments, i.e., control, *A. fabrum* (RB), 0.40 mM GABA, and 0.40 mM GABA + RB*,* were applied under 0Ni and 80 mg Ni/kg soil (80Ni) stress. Results showed that RB + 0.40 mM GABA caused significant improvements in shoot length (~ 13%), shoot fresh weight (~ 47%), shoot dry weight (~ 47%), root length (~ 13%), root fresh weight (~ 60%), and root dry weight (~ 15%) over control under 80 Ni toxicity. A significant enhancement in total chlorophyll (~ 14%), photosynthetic rate (~ 17%), stomatal CO_2_ concentration (~ 19%), leaves and roots N (~ 10 and ~ 37%), P (~ 18 and ~ 7%) and K (~ 11 and ~ 30%) concentrations, while a decrease in Ni (~ 83 and ~ 49%) concentration also confirmed the effectiveness of RB + 0.40 mM GABA than control under 80Ni. In conclusion, *fabrum* + 0.40 mM GABA can potentially alleviate the Ni toxicity in fenugreek plants. The implications of these findings extend to agricultural practices, environmental remediation efforts, nutritional security, and ecological impact. Further research is recommended to elucidate the underlying mechanisms, assess long-term effects, and determine the practical feasibility of using *A. fabrum* + 0.40GABA to improve growth in different crops under Ni toxicity.

## Introduction

Among different biotic and abiotic stresses^1–5^ nickel (Ni) accumulation in agricultural areas is a serious issue. The intentional and unintentional discharge of untreated Ni-contaminated sewage sludge and industrial effluents resulted in its accumulation in soil^6–8^. Its toxicity in plants can hinder seed germination and growth, thus decreasing crop biomass production^9,10^. Furthermore, Ni toxicity leads to chlorosis, necrosis, and interference with vital physiological processes such as photosynthesis and transpiration, resulting in oxidative damage in plants^11–13^. Rhizobacteria inoculation can play a crucial role in overcoming this critical problem.

The rhizosphere has various soil microbes, i.e., rhizobacteria and fungi, which benefit plant growth^14–16^. These rhizobacteria and fungi play a direct or indirect role in enhancing both plant growth and soil health^14,15,17–20^. In soil, rhizobacteria secrete enzymes that aid in mobilizing fixed nutrients, thus improving their uptake by the plant^21^. Numerous studies reported that rhizobacteria, which promote plant growth, can also mitigate the toxicity of heavy metals such as Ni. Certain rhizobacteria release organic secretions resulting from the chelation of heavy metals, thereby reducing their uptake by the plants^22^.

Applying γ-Aminobutyric Acid (GABA) on plants can positively affect their growth and overall health under stress conditions^23–27^. It is a non-protein amino acid that is a signaling molecule in plants and plays a vital role in various physiological processes^25^. GABA plays a pivotal role as an osmoprotectant, cellular osmotic balance and shields plants from the adverse impacts of elevated salt concentrations^28^. Additionally, its capabilities extend to scavenging reactive oxygen species, stabilizing membranes, and modulating ion transport, ultimately preserving cellular integrity and function^29^.

Fenugreek is a plant native to Southern Asia, Europe, and the Mediterranean, used in food and traditional medicine in the Indo-Pak subcontinent^30–32^. It has around 260 species, 18 of which have been identified. The plant is self-pollinated and known for treating various conditions^31^. Fenugreek seeds are rich in vitamins and regulate blood sugar and cholesterol^33^. However, the toxicity of heavy metals significantly decreases its growth^34–41^.

Hence, recognizing the significance of rhizobacteria inoculation and GABA, the present study was designed to investigate the impact of *A. fabrum* (RB) and GABA on the growth, nutrient uptake, and biochemical attributes of fenugreek plants, both with and without nickel (Ni) toxicity. The objective was to assess the efficacy of sole and combined applications of RB and recommended doses of GABA in promoting fenugreek growth under normal conditions and Ni stress. This study addresses a critical knowledge gap regarding using RB inoculation and GABA foliar application to alleviate Ni toxicity. It is hypothesized that RB with GABA may exhibit considerable potential to promote fenugreek plant growth under normal conditions and Ni toxicity.

## Materials and methods

### γ-Aminobutyric acid

The γ-Aminobutyric acid (GABA) of 0.40 mM was prepared using analytical grade salt purchased from a local certified Sigma Aldrich dealer. The detailed description includes GABA = Product Number: A2129; Batch Number: BCCJ0874; Color: White; Appearance = Powder; Sigma-Aldrich. This concentration of 0.40 mM GABA was selected based on^20^.

### Soil collection and pot filling

The soil used in the experiment was sourced from a plough layer of the bank of Chenab River, Multan, Punjab, Pakistan (30° 18′ 22.4″ N and 71° 26′ 16.3″ E). Finally, the soil was sieved through a 2 mm sieve. Each pot was filled with 5 kg of soil at a bulk density of 1.20 g cm^−3^.

### Seeds sterilization

Fenugreek seeds (Qasuri Methi) were purchased from a certified seed dealer authorized by the Government of Punjab, Pakistan. The seeds were sterilized with a 5% sodium hypochlorite solution, followed by three washes using 95% ethanol. Subsequently, the seeds underwent three additional washes with sterilized deionized water to eliminate any residual sterilizing agents. Each pot containing 5 kg of soil was sown with 20 seeds. After germination, thinning was performed to maintain five seedlings in each pot.

### Seeds inoculation with rhizobacteria and GABA foliar

Inoculating fenugreek seeds with *A. fabrum* (RB) was conducted using peat and 10% sugar solution as sticky material. For 50 g seeds, 10 g peat was used while 10 ml of sugar solution was added. After inoculation, the seeds were allowed to dry under controlled conditions to ensure proper adhesion of the inoculum to the seed surface. The characteristics of rhizobacteria are provided in Table 1. The solution was made in deionized, sterilized water. The solution of 0.40 mM GABA was applied as foliar at a rate of 25 mL per pot five times (at 7, 14, 21, 28, and 35 days after transplantation).

**Table 1:** Characterization of rhizobacteria *A. fabrum* (RB).

Attributes	Units	Values	References
IAA without L-tryptophan	(µg/mL)	9.12 ± 0.21	^42^
IAA with L-tryptophan	(µg/mL)	78.6 ± 1.27	
ACC deaminase	(µmol α-ketobutyrate nmol mg^−1^ protein h^−1^)	211.1 ± 9.46	^43^
Phosphorus solubilization	(µg/mL)	10.11 ± 2.33	^44^
Potassium solubilization	(µg/mL)	20.17 ± 2.27	^45^

### Experimental design and treatments

The experiment followed a completely randomized design (CRD) with a factorial arrangement of treatments. The treatment plan includes control (no treatment of rhizobacteria and GABA), inoculation of rhizobacteria *A. fabrum* (RB), 0.40 mM GABA as foliar application and RB + 0.40 mM GABA. Each treatment was replicated three times with and without Ni toxicity (80 mg Ni/ kg soil).

### Ni toxicity development

To develop 80 mg Ni concentration/kg soil, nickel (II) sulfate hexahydrate was purchased from Sigma's certified dealer in Multan, Punjab, Pakistan. The characteristics of salt include product Number N4882-BULK, batch Number 0000315945, CAS Number 10101-97-0, molecular formula of NiSO_4_·6H_2_O and formula weight 262.85 g/mol, form powder and crystal. Initially, salt was mixed in the soil manually, and the soil's moisture was maintained at 40% field capacity w/w. After that, it was incubated and mixing continuously after each 5 days. The moisture was maintained using a YIERYI 4 in 1 moisture meter (Shenzhen, Guangdong Province, China) for 25 days.

### Fertilizer

To meet the nutrient requirements of fenugreek, nitrogen (N) and phosphorus (P) were applied at recommended rates of 5 kg N (0.031 g N/pot) and 8 kg P_2_O_5_ /acre (0.05 g N/pot), respectively^19^. Urea was used for nitrogen application, and single superphosphate fulfilled the phosphorus requirement.

### Harvesting and data collection

Data was collected after 50 days of sowing when the plants were harvested. Fresh shoots and root weights were recorded after the harvest using a digital balance. To determine the shoot and root dry weights, samples were oven-dried at 65 °C for 72 h until a constant weight was achieved.

### Chlorophyll contents and carotenoids

We followed the standard protocol of Arnon to assess the chlorophyll a, chlorophyll b, and total chlorophyll contents in fresh leaves^46^. The extraction process involved using an 80% acetone solution, and the final absorbance was taken at 663, 645, and 480 nm.

### Gas exchange attributes

The leaf gas exchange parameters were assessed using an infrared gas Analyzer (specifically, the CI-340 Photosynthesis system by CID, Inc. USA)^47^.

### Antioxidants

To assess superoxide dismutase (SOD) activity, the inhibition of nitro blue tetrazolium (NBT) reduction was noted at 560 nm^48^. Peroxidase (POD) activity was determined by observing the oxidation of guaiacol at 470 nm^49^. Catalase (CAT) activity was assessed by monitoring the enzyme’s decomposition of hydrogen peroxide (H_2_O_2_) at 240 nm^50^. For ascorbate peroxidase (APx) activity, ascorbate oxidation in the presence of H_2_O_2_ was monitored at 290 nm^51^. To determine the malondialdehyde (MDA), the thiobarbituric acid (TBA) colored complex was assessed at 532 nm.

### Electrolyte leakage (EL)

Uniform size (1 cm) leaf pieces of 1 g were placed in test tubes containing 20 ml of deionized water. The test tubes were incubated at a constant temperature of 25 °C for 24 h, and then electrical conductivity (EC1) was measured using an EC meter. After that, test tubes were subjected to a water bath at a temperature of 120 °C for 20 min, and the second electrical conductivity (EC2) was recorded^52^.

### N, P, K and Ni in shoot and root

The digestion of shoot and root samples was done using sulfuric acid and digestion mixture^53^. The nitrogen content was determined using a modified micro-Kjeldahl method^54^. Phosphorus (P) and potassium (K) were analyzed after the digestion of shoot and root samples using a di-acid mixture^55^. The flamephotometer measured potassium concentration in the digested shoot and root samples. Meanwhile, the phosphorus concentration was quantified at 420 nm using the yellow color method, utilizing a spectrophotometer^51^. An atomic absorption spectrophotometer was used to measure Ni concentration in the di-acid digested shoot and root samples^56^.

### Statistical analysis

The collected data underwent standard statistical analysis for comparison. A two-way ANOVA was applied to assess the significance of treatments. The Tukey test was utilized for pairwise comparison of treatments with a significance level set at *p* ≤ 0.05. Additionally, cluster plot convex hull, hierarchical cluster plot, and Pearson correlation were performed using OriginPro software^57^ to further explore patterns and relationships in the data.

### Ethics approval and consent to participate

We all declare that manuscript reporting studies do not involve any human participants, human data, or human tissue. So, it is not applicable.

### Study protocol must comply with relevant institutional, national, and international guidelines and legislation

Our experiment follows the with relevant institutional, national, and international guidelines and legislation.

## Results

### Shoot attributes

**Figure 1:**
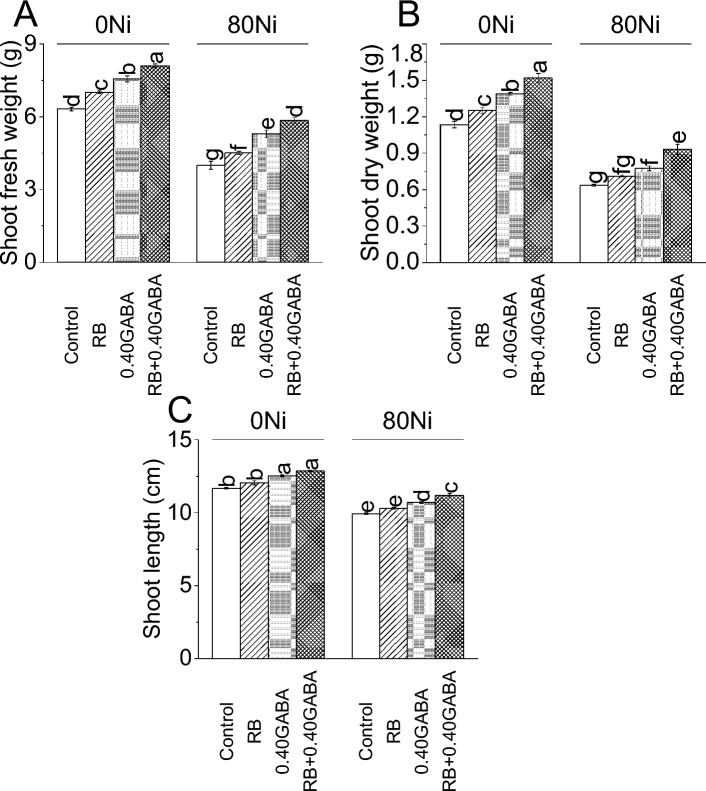
Impact of *A. fabrum* (RB) and GABA on fenugreek shoot fresh weight (**A**), dry weight (**B**), and shoot length (**C**) under 0Ni and 80 mg Ni/kg soil. The bars (n = 4) ± SE showed different letters for treatments significance at *p* ≤ 0.05; Tukey test.

Inoculation of *A. fabrum* (RB) led to an 11.04%, 0.40GABA caused 19.75%, and RB + 0.40GABA resulted in a 28.18% increase in shoot fresh weight over the control at 0Ni. Under 80 Ni stress, RB showed 13.02%, 0.40GABA 32.48% and RB + 0.40GABA 46.50% increase in shoot fresh weight than control (Fig. 1A).

In the case of 0Ni, inoculation of RB led to a 10.35% increase in shoot dry weight compared to control. Applying 0.40GABA resulted in 22.47%, while RB + 0.40GABA showed a 33.92% increase in shoot dry weight compared to the control under 0Ni. At 80Ni stress, over control, applying RB showed a 12.20% improvement in shoot dry weight. Adding 0.40GABA and RB and 0.40GABA resulted in a 22.05% and 46.85% increase from the control at 80Ni stress (Fig. 1B).

Treatment RB*,* 0.40GABA and *fabrum* + 0.40GABA caused 3.23, 7.25 and 10.16% improvement in shoot length from control under 0Ni. At 80 Ni stress, 3.55, 7.65 and 12.68% enhancement in shoot length was noted where RB*,* 0.40GABA and RB + 0.40GABA were applied respectively over control (Fig. 1C).

### Root attributes

**Figure 2:**
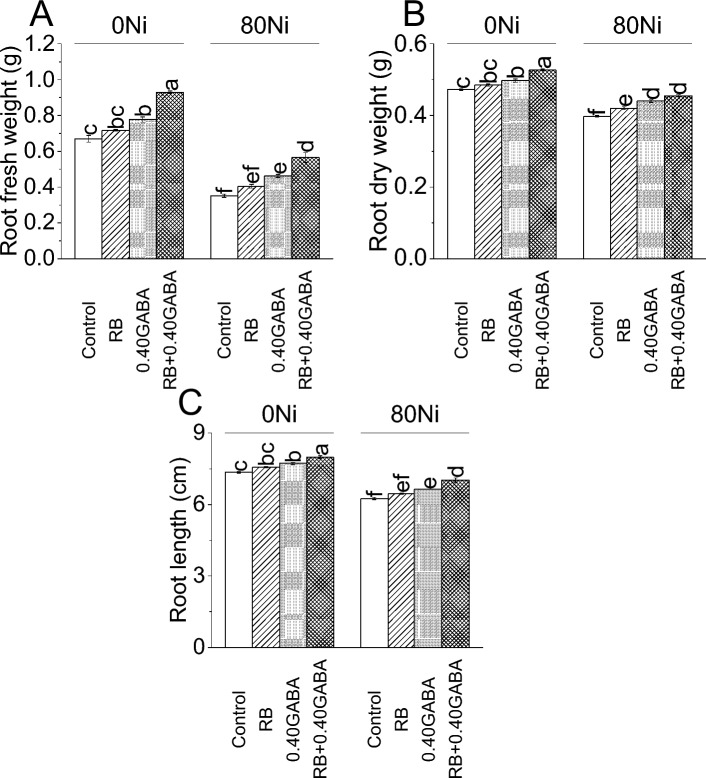
Impact of *A. fabrum* (RB) and GABA on fenugreek root fresh weight (**A**), dry weight (**B**) and root length (**C**) under 0Ni and 80 mg Ni/kg soil. The bars (n = 4) ± SE showed different letters for treatments significance at *p* ≤ 0.05; Tukey test.

Applying RB resulted in 7.09%, 0.40GABA caused 16.04%, while RB + 0.40GABA showed a 38.81% increase in root fresh weight than the control at 0Ni. In the case of 80Ni, RB showed a 14.89% increase in root fresh weight than control. Applying 0.40GABA resulted in 31.21%, while RB + 0.40GABA had a 60.28% increase in root fresh weight compared to control under 80Ni stress (Fig. 2A).

For root dry weight, an increase of 2.65, 5.29 and 11.64% was observed in RB, 0.40GABA and RB + 0.40GABA over control under 0Ni. Under 80Ni stress, compared to control, a 5.66% increase in root dry weight was noted in RB, 10.69% in 0.40GABA and 14.47% in RB + 0.40GABA (Fig. 2B).

Adding RB caused 2.95%, while 0.40GABA resulted in a 5.03% increase in root length over the control group under 0Ni. Treatment RB + 0.40GABA showed an 8.60% enhancement in root length compared to the control at 0Ni. Under 80Ni stress, an increase was noted in root length where RB (3.28%), 0.40GABA (6.23%) and RB + 0.40GABA (12.47%) were applied over control (Fig. 2C).

### Chlorophyll a, b, and total

**Figure 3:**
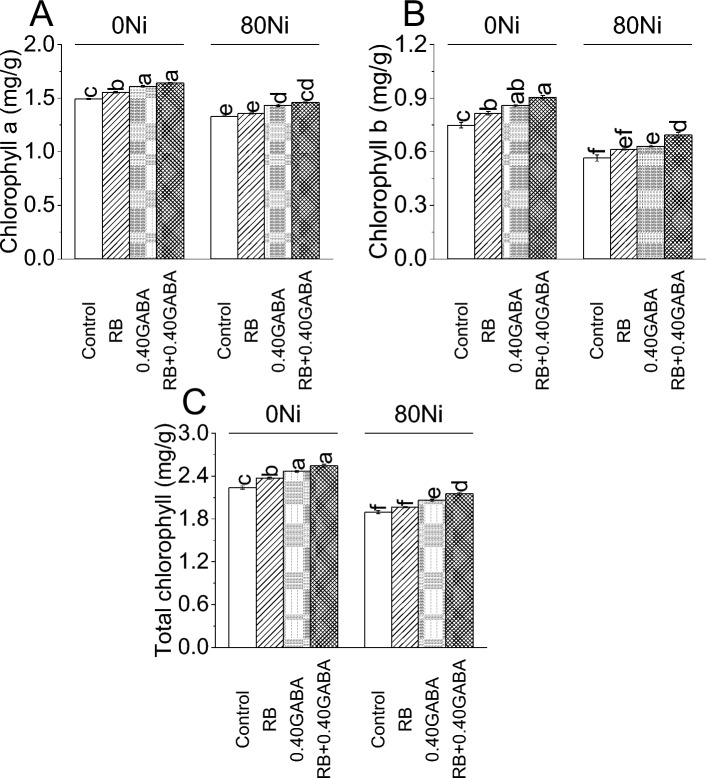
Impact of *A. fabrum* (RB) and GABA on fenugreek chlorophyll a (**A**), chlorophyll b (**B**), and total chlorophyll (**C**) under 0Ni and 80 mg Ni/kg soil. The bars (n = 4) ± SE showed different letters for treatments significance at *p* ≤ 0.05; Tukey test.

Results showed that RB increased chlorophyll a content by 4.19% while 0.40GABA by 7.87% compared to control at 0Ni. Treatment RB + 0.40GABA showed a 9.88% improvement in chlorophyll a content than control under 0Ni. Inoculation of RB increased chlorophyll a content (1.36%) than control under 80 Ni stress. However, 0.40GABA showed 7.71%, and RB + 0.40GABA showed a 9.77% improvement in chlorophyll a content compared to the control under 80Ni stress (Fig. 3A).

At 0Ni, 0.40GABA caused 14.72%, RB showed 9.03%, and RB + 0.40GABA resulted in a 21.07% increase in chlorophyll b content from control. At 80Ni stress, applying RB resulted in 8.41%, 0.40GABA 11.50% and RB + 0.40GABA showed a 22.57% increase in chlorophyll b content from control (Fig. 3B).

In the case of total chlorophyll content, an increase of 5.80, 10.16 and 13.62% was observed in RB, 0.40GABA and RB + 0.40GABA, respectively, compared at 0Ni. Under 80 Ni stress, RB caused 3.83%, 0.40GABA 8.84% and RB + 0.40GABA 13.59% enhancement in total chlorophyll over control with 80Ni stress (Fig. 3C).

### Gas exchange attributes

**Figure 4:**
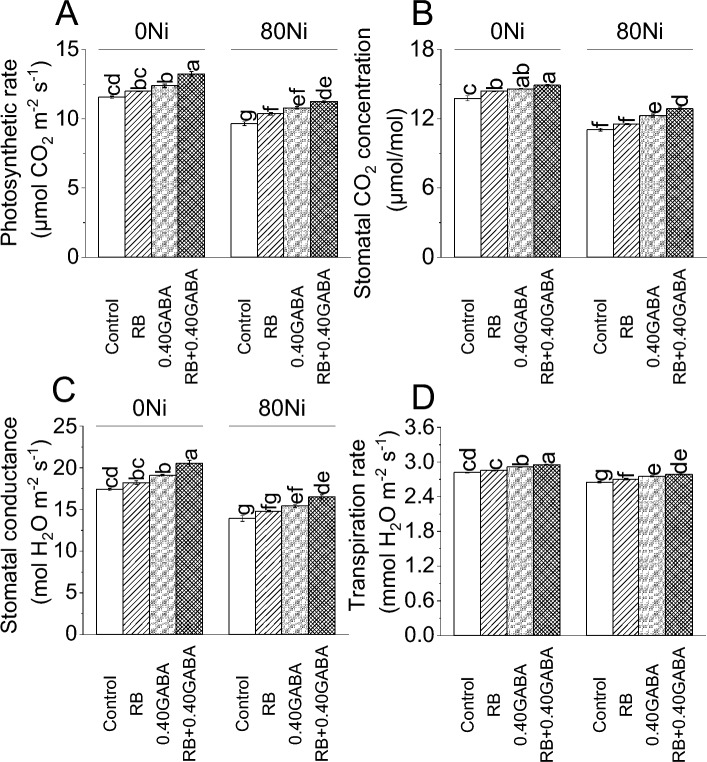
Impact of *A. fabrum* (RB) and GABA on fenugreek photosynthetic rate (**A**), stomatal CO_2_ concentration (**B**), stomatal conductance (**C**) and transpiration rate (**D**) under 0Ni and 80 mg Ni/kg soil. The bars (n = 4) ± SE showed different letters for treatments significance at *p* ≤ 0.05; Tukey test.

Inoculating RB increased the photosynthetic rate by 3.65% while 0.40GABA treatment by 7.13% compared to control under 0Ni. It was noted that RB + 0.40GABA caused a 14.41% enhancement in photosynthetic rate over control at no Ni toxicity. RB caused 7.55%, 0.40 GABA 11.98% and RB + 0.40GABA 16.78% increase in photosynthetic rate from control under 80 Ni stress (Fig. 4A).

In the case of stomatal CO_2_ concentration, a 4.79% increase was found in RB, 9.89% in 0.40 GABA and 18.09% in RB + 0.40 GABA compared to control under 0Ni. In 80Ni stress, RB caused 6.39%, 0.40 GABA 10.86%, and RB + 0.40 GABA 18.54% increase in stomatal CO_2_ concentration than control (Fig. 4B).

At 0Ni, RB increased stomatal conductance by 4.43% compared to the control. On the other hand, 0.40 GABA caused an enhancement of 5.95%, while RB with 0.40 GABA showed an 8.40% increase over the control under 0Ni. Inoculating RB increased stomatal conductance by 4.52%, 0.40 GABA by 11.03% and RB + 0.40 GABA by 16.62% over the control under 80Ni stress (Fig. 4C).

It was noted that applying RB resulted in 5.20%, 0.40GABA 11.08% and RB + 0.40GABA 14.64% increase in the transpiration rate than control under 0Ni. In the case of 80Ni stress, RB resulted in 1.98%, 0.40GABA 3.87% and RB + 0.40GABA 5.19% increase in transpiration rate compared to control under 80Ni stress (Fig. 4D).

### Antioxidants

**Figure 5:**
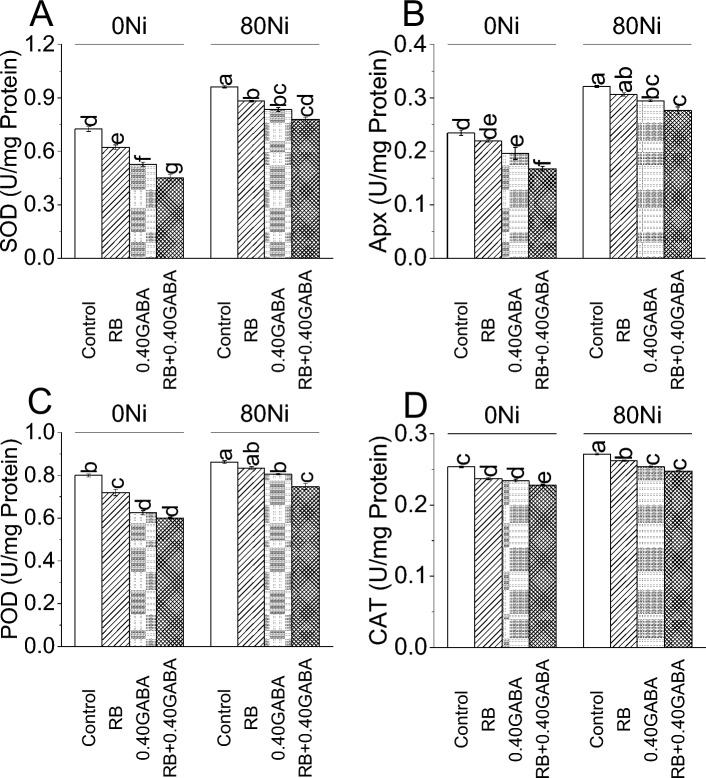
Impact of *A. fabrum* (RB) and GABA on fenugreek SOD (**A**), APx (**B**), POD (**C**), and CAT (**D**) under 0Ni and 80mg Ni/kg soil. The bars (n = 4) ± SE showed different letters for treatments significance at *p* ≤ 0.05; Tukey test.

Treatments RB caused 16.47% while 0.40 GABA showed a 37.44% decline in SOD activity under 0Ni. It was noted that RB + 0.40 GABA resulted in a 60.22% decrease in SOD activity where 0Ni was present. Under 80 Ni stress, RB reduced SOD activity by 9.07%, 0.40 GABA by 15.27% and RB + 0.40 GABA by 23.40% over control (Fig. 5A).

**Figure 6:**
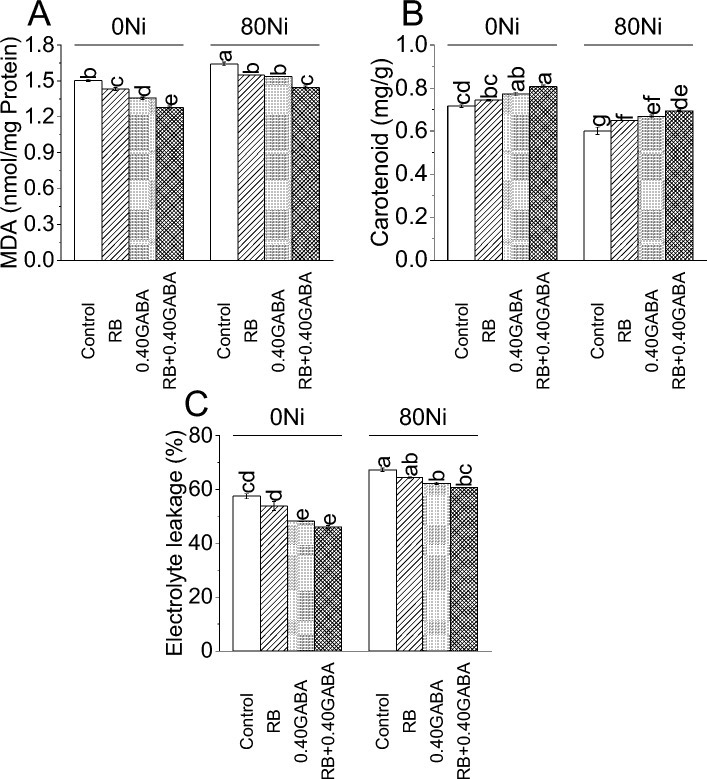
Impact of *A. fabrum* (RB) and GABA on fenugreek MDA (**A**), carotenoids (**B**), and electrolyte leakage (**C**) under 0Ni and 80mg Ni/kg soil. The bars (n = 4) ± SE showed different letters for treatments significance at *p* ≤ 0.05; Tukey test.

In the case of Apx activity, RB showed a 6.83% decrease, while 0.40 GABA showed a 19.64% decrease over control under 0Ni. Applying RB with 0.40 GABA resulted in a 39.79% decrease compared to the control at 0Ni. At 80Ni stress, compared to control Apx activity, it decreased by 4.98%, 9.08%, and 16.27%, where RB, 0.40 GABA, and RB + 0.40 GABA were applied, respectively (Fig. 6B).

Under 0Ni, RB decreased 11.11% in POD activity over control. Adding 0.40GABA and RB + 0.40GABA, treatments also reduced the POD activity by 28.00% and 33.33% compared to the control at 0Ni. In the case of 80Ni stress, RB showed a 3.29%, 0.40GABA 7.14%, and RB + 0.40GABA 15.38% decline in POD activity from control (Fig. 5C).

Applying RB resulted in 6.95%, while 0.40 GABA showed an 8.21% decline in CAT activity compared to the control group in 0Ni. Treatment RB + 0.40 GABA showed an 11.17% decrease in CAT activity compared to the control under 0Ni. Results showed that RB caused 3.53% 0.40 GABA 7.00% and RB + 0.40 GABA 9.48% decrease in CAT activity over control under 80Ni stress (Fig. 5D).

### Malondialdehyde (MDA), Ascorbate peroxidase (Apx) and Electrolyte leakage

Inoculation of RB reduced 5.06%, and 0.40 GABA caused an 11.07% decline in MDA compared to the control under 0Ni. However, RB + 0.40 GABA showed a 17.81% decrease in MDA than the control at 0Ni. On the other hand, RB resulted in 5.97%, 0.40 GABA showed 6.85%, and RB + 0.40 GABA caused a 13.67% decrease in MDA than control under 80Ni stress (Fig. 6A).

A significant increase of 3.91%, 0.76%, and 12.81% in carotenoids was observed in RB, 0.40 GABA, and RB + 0.40 GABA over control at 0Ni, respectively. In 80Ni stress, applying RB caused 8.07%, 0.40 GABA 11.19%, while RB + 0.40 GABA 15.47% increase in carotenoids than control (Fig. 5B).

A decline of 6.79%, 19.30%, and 45.95% for electrolyte leakage was noted in RB, 0.40 GABA, and RB + 0.40 GABA over control under 0Ni. Results showed that RB, 0.40 GABA, and RB + 0.40 GABA also caused a significant decrease of 4.61%, 8.11%, and 10.88% in electrolyte leakage, respectively, compared to control in 80 Ni stress (Fig. 6C).

### Shoot N, P, K and Ni concentration

Under 0Ni and 80 Ni stress, RB and 0.40 GABA increased shoot N compared to the control. The RB + 0.40 GABA improved by 10.34 and 9.62% shoot N over control under 0Ni and 80 Ni stress, respectively. Similar results were also noted in shoot P, where RB and 0.40 GABA caused a significant enhancement in shoot P compared to the control group at 0Ni and 80 Ni stress. However, RB + 0.40 GABA showed 14.29 and 17.65% enhancement in shoot P compared to control at 0Ni and 80 Ni stress, respectively. In the case of shoot K, treatment RB + 0.40 GABA performed significantly better, showing an increase of 7.94 and 10.53% than control in 0Ni and 80 Ni stress, respectively. At 80 Ni stress, RB and 0.40 GABA treatments showed a decrease in Ni concentration. Treatment RB + 0.40 GABA under 80 Ni stress performed significantly best by reducing Ni concentration over control (Table 2).

**Table 2:** Effect of treatment on N, P, K, and uptake of Ni concentration of shoot and root.

Stress	Treatment	Shoot N (%)	Shoot P (%)	Shoot K (%)	Leaves Ni (µg/g)
0Ni	Control	0.87c	0.42c	0.63c	0.20de
	RB	0.88c	0.45b	0.64bc	0.18f
	0.40 GABA	0.92b	0.47ab	0.66b	0.14g
	RB + 0.40GABA	0.96a	0.48a	0.68a	0.10h
80 Ni stress	Control	0.78f	0.34f	0.57f	1.29a
	RB	0.80e	0.36e	0.59e	0.27b
	0.40 GABA	0.81e	0.38e	0.61de	0.23c
	RB + 0.40GABA	0.85d	0.40d	0.63cd	0.22cd

Stress	Treatment	Root N (%)	Root P (%)	Root K (%)	Root Ni (µg/g)
0Ni	Control	0.83cd	0.068c	0.23c	0.64e
	RB	0.85bc	0.071b	0.25b	0.12g
	0.40 GABA	0.89ab	0.073b	0.26ab	0.54f
	RB + 0.40GABA	0.91a	0.076a	0.27a	0.31h
80 Ni stress	Control	0.62g	0.061f	0.16f	10.58a
	RB	0.71f	0.062ef	0.18e	7.54b
	0.40 GABA	0.75ef	0.063de	0.19e	6.14c
	RB + 0.40GABA	0.79de	0.065d	0.21d	5.35d

Values are an average of 4 replicates. Different letters showed significant changes at *p* ≤ 0.05, Tukey Test. *A. fabrum* = RB.

### Root N, P, K and Ni concentration

Results showed a significant improvement in root N, P, and K, while a decline in Ni concentration was observed where RB and 0.40 GABA were treated under 0Ni and 80 Ni stress. It was noted that RB + 0.40 GABA showed an increase in root N (9.64 and 37.10%), P (11.76 and 6.56%), and K (17.39 and 25.00%) while a decrease in Ni compared to control at 0Ni and 80 Ni stress respectively.

The first two principal components accounted for substantial variance in the data, capturing 98.08% and 0.64%, respectively. Most data points, characterized by 0Ni, form a tight cluster in the lower-right quadrant of the plot. These data points are represented by scores on PC1 ranging from approximately 0.25 to 7.39 and scores on PC2 ranging from approximately −0.38 to 0.12.

**Figure 7:**
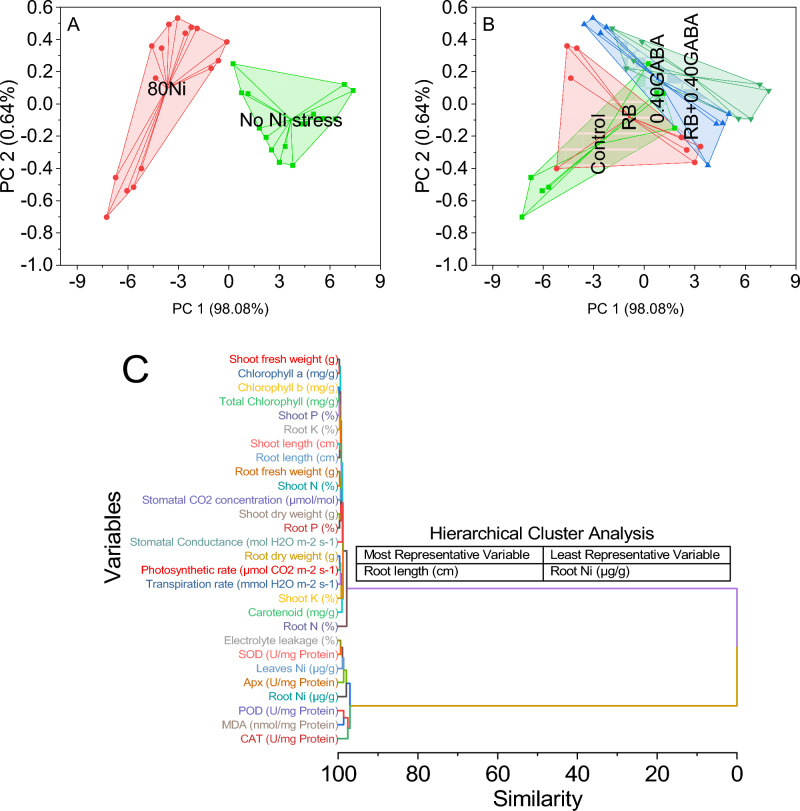
Cluster plots convex hull for applied Ni stress (**A**), different treatments (**B**), and hierarchical cluster plot (**C**) for the studied attributes.

In contrast, a smaller group of data points, labeled as 80Ni, form a separate cluster in the upper-left quadrant of the plot. These data points exhibit scores on PC1 ranging from approximately −7.25 to −0.12 and scores on PC2 ranging from approximately −0.70 to 0.39 (Fig. 7A). A smaller cluster labeled 0.40GABA is also located in the plot’s lower center. The data points in this cluster have scores on PC1 ranging from approximately −3.57 to 5.59 and scores on PC2 ranging from approximately −0.38 to 0.39. Lastly, a cluster labeled RB + 0.40GABA is in the upper-right quadrant of the plot. The data points in this cluster display scores on PC1 ranging from approximately −1.90 to 7.39 and scores on PC2 ranging from approximately −0.40 to 0.53 (Fig. 7B).

Chlorophyll b (mg/g) and total chlorophyll (mg/g) showed a high similarity of 0.22735, suggesting a close relationship between these two chlorophyll-related variables. Similarly, shoot P (%) and chlorophyll a (mg/g) demonstrated a significant similarity of 0.29596, indicating a potential association between shoot phosphorus content and chlorophyll a level. Shoot length (cm) and root length (cm) exhibited a similarity of 0.34924, indicating their linkage in terms of plant morphology. Similarly, root fresh weight (g) and shoot nitrogen (%) showed a similarity of 0.35047, suggesting a possible correlation between root weight and shoot nitrogen content. On the other hand, some variables appeared to be significantly dissimilar to others. Carotenoid (mg/g) and stomatal conductance (mol H_2_O m^−2^ s^−1^) displayed a dissimilarity of 1.074, indicating they might be unrelated in this context (Fig. 7C).

## Discussion

### Ni toxicity

Ni contamination affected the nutrient uptake, growth, and physiology of Fengureek in our study^41^. This was obvious due to higher Ni concentration in the root and leaf (Table 2). Ashraf et al.^58^ found a noticeable decrease in growth, biomass, photosynthetic pigments, and nutrient acquisition in response to nickel toxicity^59^. An overabundance of Ni in plants can disturb various physiological processes, adversely impacting growth and development^60^. Despite its role as a crucial micronutrient in enzyme activation and nitrogen metabolism, Ni can turn toxic when present in elevated concentrations^61^. It can also disrupt enzyme functions, trigger oxidative stress, and create nutrient imbalances by interfering with the assimilation of vital elements^62^. The impairment of root growth further hampered water and nutrient absorption, leading to stunted growth, diminished biomass, and reduced crop yields^63^.

### GABA

The uptake of Ni in roots and leaves was reduced with the application of GABA in our study (Table 2)^42^. This might be because GABA is known to modulate stress responses and stimulate antioxidant systems in plants, which could mitigate the impact of nickel-induced oxidative stress and, in turn, influence the plant’s ability to cope with nickel uptake^64,65^. In response to abiotic stresses, signaling triggered by GABAs is linked to stimulating the antioxidative defense system, regulating osmotic balance, maintaining levels, and acting as a buffering agent for carbon and nitrogen metabolism^66^. The low electrolyte leakage and MDA in the current study also validated the positive functioning of GABA regarding the alleviation of Ni stress (Fig. 6). GABA can bind and sequester Ni ions, which decreases their mobility. This procedure reduces Ni buildup by stopping the transport of Ni to delicate plant organs^67^.

### *A. fabrum*

*A. fabrum* inoculation decreased the uptake of Ni and enhanced N, P, and K nutrients in the root and leaf (Table 2). This was mainly attributed to this rhizobacteria’s P and K solubilizing ability (Table 1). Enhanced uptake of essential nutrients such as nitrogen (N), phosphorus (P), and potassium (K) plays a pivotal role in promoting plant growth. Improved nutrient absorption facilitates robust metabolic processes, supports energy transfer, and enhances cell division^68^. This heightened nutrient availability synthesizes vital biomolecules, increasing photosynthetic efficiency, healthier root development, and ultimately fostering overall plant growth and productivity^69^. Matile et al.^70^ proposed that elevated ethylene concentrations due to abiotic stress, i.e., heavy metal toxicity^71^, lead to the breakdown of lipids in the cell wall. They posited that when ethylene meets the chlorophyllase (chlase) gene, it induces chlorophyll degradation, resulting in chlorosis. In the current study, improvement in the chlorophyll contents validated the effectiveness of RB as the rhizobacteria could secrete ACC deaminase, which was imperative in decreasing the stress ethylene^16,72^. Furthermore, phytohormones, i.e., indole-3-acetic acid (IAA), also have essential biological functions in improving the growth of plants^73^.

### Combined effects of *A. fabrum* and GABA

GABA can potentially improve nutrient absorption and translocation inside the plant, whereas rhizobacteria increase nutrient availability in the rhizosphere^74^. Even when Ni stress is present, this cooperative effort provides an adequate delivery of vital nutrients. It functions as a stress reliever, lowering the adverse effects of Ni stress on fenugreek, while rhizobacteria prime the plant’s defense systems and improve stress tolerance^75^. Rhizobacteria and GABA together, the plant’s adaptive responses may be enhanced through overexpression of stress tolerance and growth promotion genes^76^.

## Conclusions

In conclusion, the combination of *A. fabrum* + 0.40 mM foliar GABA (0.40GABA) proved highly effective in improving shoot and root growth parameters, chlorophyll content, and gas exchange attributes. Moreover, applying *A. fabrum* + 0.40GABA can also improve antioxidants, N, P, and K concentration in leaves and roots while reducing Ni concentration. These findings collectively confirmed the efficacy of *A. fabrum* + 0.40GABA in alleviating Ni stress and enhancing the growth and health of fenugreek plants exposed to Ni toxicity.

## Data Availability

All data generated or analyzed during this study are included in this published article.
